# A Morphometric Study on the Dimensions of the Vertebral Canal and Intervertebral Discs from Th1 to S1 in Cats and Their Relevance for Spinal Diseases

**DOI:** 10.3390/vetsci11090429

**Published:** 2024-09-13

**Authors:** Jessica Richter, Christoph K. W. Mülling, Nicole Röhrmann

**Affiliations:** Faculty of Veterinary Medicine, Institute of Veterinary Anatomy, Histology and Embryology, Leipzig University, An den Tierkliniken 43, D-04103 Leipzig, Germany; christoph.muelling@vetmed.uni-leipzig.de (C.K.W.M.); nicole.roehrmann@vetmed.uni-leipzig.de (N.R.)

**Keywords:** cat, morphometry, vertebral canal, spine, intervertebral disc

## Abstract

**Simple Summary:**

The vertebral canal dimensions are crucial for spinal health, as degenerative changes can significantly affect the spinal cord. Compression of spinal nerves at the lumbosacral junction causes cauda equina syndrome (CES), more common in dogs than cats. With regard to the limited information on feline vertebral canal dimensions, this study examined 50 cats, measuring the interpedicular (ID) and midsagittal (SD) diameters as well as the intervertebral disc width (IVDW) in 28 of these animals. The region between the first thoracic (Th1) and the first sacral vertebra (S1) was considered using dissected cats. All cats showed a notable narrowing of the spinal canal from L6 to S1 with the narrowest point at S1. The widest parts of the vertebral canal correspond with the spinal cord enlargements. IVDW was found to be fairly consistent up to Th10–Th11, with the widest discs present at L7–S1 in 95.65% of cats. These data aim to understand potential correlations between the vertebral canal dimensions and the IVDW in terms of predispositions to spinal diseases in cats, especially compressive myelopathies. Further studies should be conducted to investigate the influence of age, sex and breed.

**Abstract:**

As part of the spine, the vertebral canal represents a central structure protecting the spinal cord running within it. Since alterations to the spinal canal and adjacent structures can have a significant impact on the spinal cord, knowledge of the physiological vertebral canal dimensions is essential. Compression of spinal nerves at the lumbosacral junction is the primary cause of cauda equina syndrome (CES). Although CES is common in dogs, it is rarely documented in cats. Given the lack of information on normal vertebral canal dimensions in cats, it is necessary to collect data and verify currently used measurements, to determine if and to what extent comparisons with dogs are valid. In 50 cats, interpedicular (ID) and midsagittal (SD) diameters were examined from the first thoracic (Th1) to the first sacral vertebra (S1). In 28 of these animals, the intervertebral disc width (IVDW) was measured. All data were gathered through gross anatomical dissection of the cats. Significant lumen reduction was evident in all cats from L6 to S1 with the narrowest point at S1. Narrowings were also found in the thoracic spine. The widest points coincide with the spinal cord enlargements. IVDW shows relatively constant values up to Th10–Th11 and peaks at L7–S1 in 95.65% of cats. While distinct similarities to dogs were observed, differences exist. The findings allow conclusions as to whether relations between the parameters and resulting predispositions to pathological changes can be derived. This could help the understanding of the pathogenesis of feline spinal diseases, particularly compressive myelopathies. Further studies are necessary to investigate the impact of age, sex and breed.

## 1. Introduction

The vertebral canal and spinal cord can be divided into five segments because of their embryological development and location. These comprise the cervical (C), thoracic (Th), lumbar (L), sacral (S) and caudal (Cd) segments. As a result of the seeming spinal cord ascent, the segments of the spinal cord shift cranially in relation to the corresponding vertebral bodies [1,2]. Because of this, the caudal spinal cord segments do not correlate with the vertebral bodies of the same name. Consequently, in felines, the segments L4–Cd5 are localized at the level of the vertebrae L4–S1 [3]. 

In cats, the tapering caudal part of the spinal cord, the conus medullaris, is usually located in the region of L7, respectively S1. In 90% of adult cats, the conus medullaris ends at the level of the sacrum, whereas the lumbosacral spinal cord is noticeably shortened in dogs [3]. This anatomical variation between the two species results in a closer correlation between the vertebrae and their respective spinal cord segments in cats. Therefore, local compression injuries have less impact on the spinal cord and the nerve roots located there than lesions in the same area in dogs [3]. The cauda equina arising from the conus medullaris is formed by the paired caudal spinal nerves of the spinal cord segments L6–S1 through Cd5–Cd8 and runs caudally to the tail [3,4]. In dogs, the vertebral canal widens at the transition from the cervical to the thoracic spine as well as in the region of the lumbar spine. In contrast, at the level of the sacrum, it narrows and tapers to the first caudal vertebrae where it finally ends [5]. It has already been discovered that the diameters of the vertebral canal vary depending on the breed and age of dogs [6,7]. One study found a correlation between the localization of the widest point in the vertebral canal and the presence of transitional vertebrae in dogs [8]. In further studies, the size variations over the length of the vertebral canal have already been established for dogs [9,10]. However, comparable studies in cats are lacking so far and little is currently known about the common dimensions of the feline vertebral canal [11].

A further integral part of the spinal cord system are the intervertebral discs. These connecting structures link the adjacent bodies of two vertebrae from the 3rd cervical vertebra through an intervertebral symphysis and represent approximately a quarter of the total length of the spine [12].

This work focuses on the dimension changes in the feline vertebral canal. Particular attention was paid to the localizations of the narrowest points and the results were compared with findings in dogs [8]. Specifically relevant was the question of whether significant correlations exist between the dimensions of the vertebral canal and predisposed localizations for pathologies in cats, such as thoracic or lumbosacral vertebral canal stenosis. One study has already shown a correlation between the presence of transitional vertebrae and lumbosacral stenosis [13]. The latter may show clinical signs of cauda equina syndrome (CES), which is initially characterized by lumbosacral pain associated with a decrease in activity [3]. Neurological malfunctions of the hindlimbs, such as proprioceptive deficits as well as fecal and urinary dysfunction up to paraparesis may occur in the advanced stage of the disease [14]. Compared to dogs, cats rarely present with CES. In cats, it is mainly due to traumatic injuries resulting in translocation, compression, or destruction of the nerve roots located there [3,15]. By comparison, lumbosacral stenoses (LSS) associated with degenerative changes are mainly listed as causal in dogs and a common reason for neurological deficits and pain in the hindlimbs [16,17]. Such diseases are infrequently seen or described in cats.

The primary cause of the marginally described stenosis of the feline vertebral canal in the thoracic region was listed as hypertrophy of the articular processes [18]. In one case, thoracic vertebral canal stenosis due to hypertrophy of a single vertebra has been reported [19]. The purpose of this work was to evaluate whether the anatomical dimensions in this area, particularly narrowings, could predispose to myelopathies in addition to these alterations. 

Since discopathies, in addition to spondyloses, can also lead to stenosis of the vertebral canal with subsequent spinal cord compression [14], this work also considers the intervertebral discs. In this regard, attention will primarily be paid to their width in the various sections as well as to associated predispositions to disc herniation.

A comparison between dogs and cats should be possible using the information gathered through the macroscopic examination and the morphometric evaluation. Moreover, it should provide detailed insights into the dimensions of the feline vertebral canal from Th1 to S1 and broaden our understanding of spinal pathologies in cats. Finally, it should be determined whether similarities can be shown between the two species and whether the findings obtained in the dog differ from the data gathered on the cat’s vertebral canal.

## 2. Materials and Methods

### 2.1. Study Population

The investigations were performed on 50 adult cats of the European Shorthair breed, disregarding weight and sex. The animals were donated by Leipzig veterinary practices to the Institute of Veterinary Anatomy for use in research and teaching. They were euthanized in accordance with animal welfare due to an inauspicious prognosis and in compliance with the applicable regulations by the attending veterinarian. The dissection as well as the measuring of the carcasses took place in the dissecting room of the Institute of Veterinary Anatomy in compliance with the applicable hygiene regulations. The animals used for the measurements had previously been employed for educational purposes.

### 2.2. Experimental Design and Measurements

**Figure 1:**
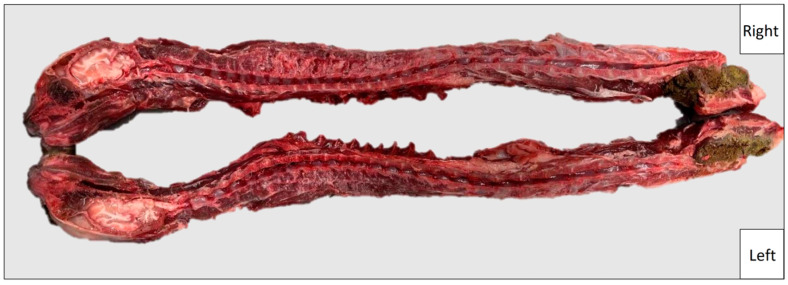
Exemplary picture of the paramedian section in a cat.

For the purpose of measuring, after preparatory resection of limbs and internal organs, cats were deep-frozen at −18 °C and subsequently cut in the median with a butcher’s band saw (Figure 1). In this context, the saw blade loss v = 0.5 mm must be taken into account for each measurement since all cuts were performed with the same saw, the same blade and by the same person. Subsequently, the spinal cord and the surrounding spinal meninges (dura mater, arachnoid membrane, pia mater) were removed from both halves in the measurement range from Th1 to S1 using scalpel and forceps. With the aid of an analog caliper, the midsagittal (SD) and interpedicular (ID) diameters were determined with an accuracy of 0.05 mm.

**Figure 2:**
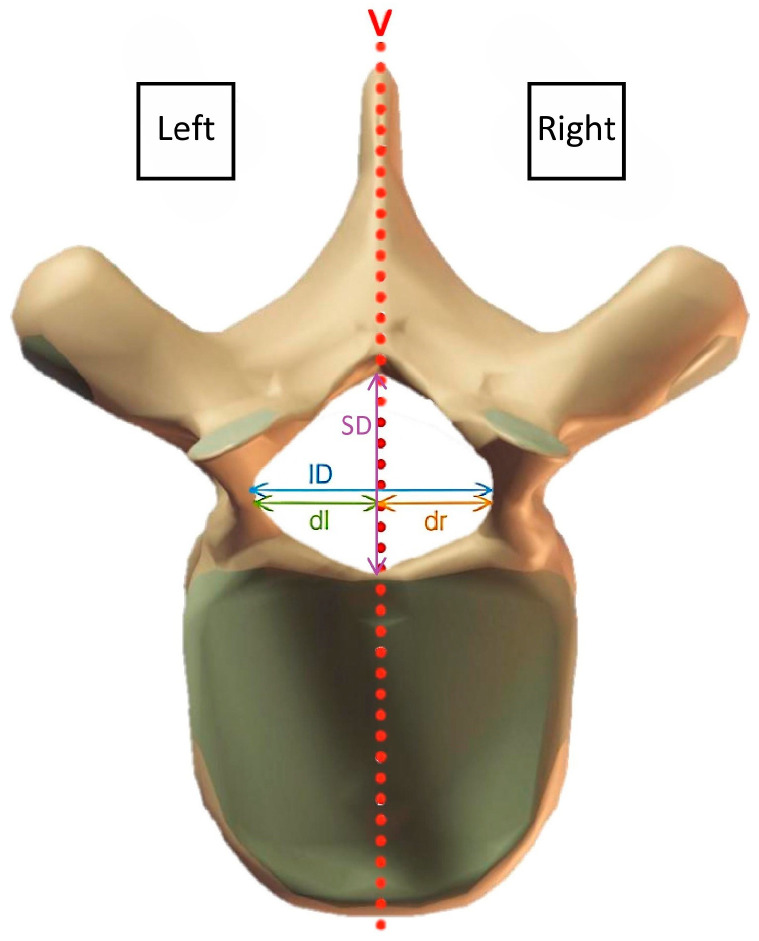
Overview of measuring parameters and their composition in transverse plane. ID = interpedicular diameter, SD = midsagittal diameter, dl = radius of the left half of the vertebra, dr = radius of the right half of the vertebra, v = saw blade loss.

To simplify the composition of the measuring parameters in the non-spherical vertebral canal, the distance from the saw line’s perpendicular to the furthest point on the inner pedicle wall of the respective vertebra is designated as the radius. Starting with the right-hand section, first, the radius of the right half of the vertebra (dr) was determined continuously from Th1 to S1. Afterward, SD was evaluated at the center of the relevant vertebral halves of the right side (SD_r_). The measurements were performed in the same way on the left half of the section to determine the radius of the left half of the vertebra (dl) as well as SD of the left side (SD_l_). To identify ID, the measured values of the right (dr) and left (dl) radius were added and complemented by the saw blade loss v (Figure 2). SD of each vertebra results from averaging the measured values from both vertebra halves. This method serves to level out cut-related variances. The following formulas can be used to describe the composition of the two parameters SD and ID:

ID = {dr + dl + v}

SD = {SD_r_ + SD_l_}/2

ID interpedicular diameter;
dr radius of the right half of the vertebra;
dl radius of the left half of the vertebra;
v saw blade loss of 0.5 mm;
SD midsagittal diameter;
SD_r_ midsagittal diameter of the right half of the vertebra;
SD_l_ midsagittal diameter of the left half of the vertebra.

In 28 of the 50 cats, the intervertebral disc widths (IVDW) were determined with an accuracy of 0.05 mm using an analog caliper. The reduced sample size resulted from the variability in the cuts, which meant that measurement of the IVDW was not always possible. In addition, a visual evaluation of the intervertebral discs was performed in all cats (*n* = 50) based on the photographic documentation. By examining the trend of IVDW in the relevant region, tendencies could be derived, and conclusions drawn about possible predilection sites for disc herniation.

Animals for which an exact median cut was not possible, were given special consideration as part of the data analysis since values could not be determined across the entire measurement range in these cases.

### 2.3. Statistical Analysis

Data analysis was performed using a statistical software package (OriginPro 2019, version 9.60, 64-bit). The values for each vertebra were tested for normal distribution using the Shapiro–Wilk test. Descriptive statistics were reported as mean with standard deviation (sd) and as median (md) and range (rg). Correlations between ID and IVDW, SD and IVDW as well as ID and SD were identified with the help of Spearman rank test using mean values. The correlation coefficient (r_s_) was determined according to Cohen [20]. Statistical significance was set to *p* < 0.05.

## 3. Results

### 3.1. Vertebral Canal Diameters (ID and SD)

**Figure 3:**
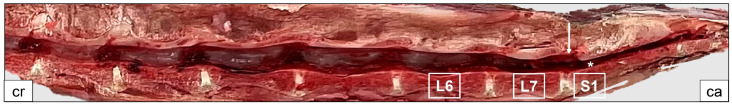
Illustration of the lumbosacral junction on right body half in one examined cat. Tapering of the vertebral canal in caudal orientation and reaching the narrowest point at the level of S1 are comprehensible. cr = cranial, ca = caudal, L6 = body of L6, L7 = body of L7, S1 = sacral basis, * narrowest point, ↓ = lumbosacral junction.

**Figure 4:**
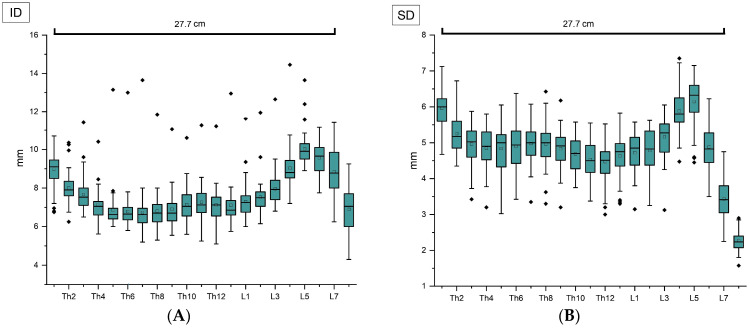
(**A**) Interpedicular diameters (ID) and (**B**) midsagittal diameters (SD) measured at the center of each vertebra from Th1 up to S1 with respect to the mean length of the vertebral canal from Th1 to L7. Mean values are shown as squares and median values as horizontal lines inside the boxes. Outliers are displayed as black diamonds outside the boxes. The height of the box represents the interquartile range (IQR), which spans from the 25th (Q1) to the 75th (Q3) percentile. The whiskers are defined as lower extreme (Q1 − 1.5 × IQR) and upper extreme (Q3 + 1.5 × IQR), representing the maximum values within 1.5 × IQR, and may appear non-symmetrical due to variations in the data distribution.

As an overview, the mean length of the vertebral canal from Th1 up to L7 was determined to be 27.7 cm. In the examinations, a clear lumen reduction was shown consistently in all 50 cats at the transition from vertebra L5 to L6 as well as from L6 to L7 and L7 to S1, for both ID and SD (Figure 3 and Figure 4).

The maximum ID values in the lumbar spine were localized at the level of L4 to L7, and between L3 and L6 when considering SD, followed by the decrease in both parameters mentioned previously. Focusing on the thoracic spine, it can be noted that a constant regression of the measured values was found from Th1 to Th4. Further narrowings of the vertebral canal diameters become apparent at the height of Th6–Th7 and from Th11 to Th13 concerning ID, and between Th9 and Th12 when considering SD. In the thoracic spine, both ID and SD show their maximum values from Th1 to Th3 in most of the cats (Figure 4).

The narrowest region of the vertebral canal was determined invariably at the level of the first sacral vertebra regarding SD and detected there in the majority of cats in terms of ID (Figure 3 and Figure 4).

### 3.2. Intervertebral Disc Width (IVDW)

**Figure 5:**
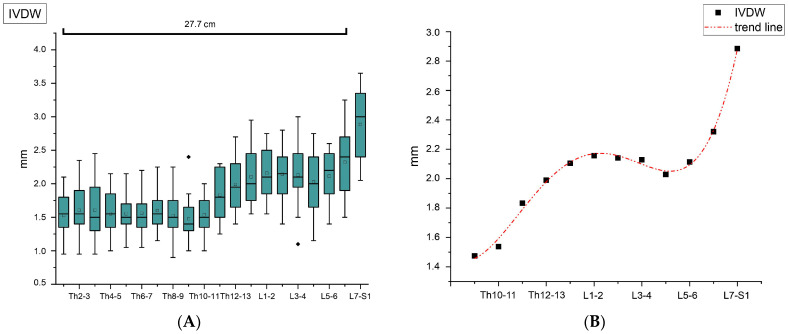
(**A**) Intervertebral disc widths (IVDW) from disc Th1–Th2 up to disc L7–S1 with respect to the mean length of the vertebral canal from Th1 to L7. Mean values are shown as squares and median values as horizontal lines inside the boxes. Outliers are displayed as black diamonds outside the boxes. The height of the box represents the interquartile range (IQR), which spans from the 25th (Q1) to the 75th (Q3) percentile. The whiskers are defined as lower extreme (Q1 − 1.5 × IQR) and upper extreme (Q3 + 1.5 × IQR), representing the maximum values within 1.5 × IQR, and may appear non-symmetrical due to variations in the data distribution. (**B**) Course of the intervertebral disc widths from disc Th9–Th10 up to disc L7–S1 shown as mean values with corresponding trend line. A distinct increase in the intervertebral disc width starting from disc Th11–Th12 and the peak at disc L7–S1 are visible.

Regarding IVDW, it is noticeable that the intervertebral discs show a comparatively constant width up to Th10–Th11 (Figure 5A). In the further course, they first become wider in caudal orientation and then continuously decrease starting from intervertebral disc L1–L2. Beginning at intervertebral disc L4–L5, a renewed increase can be observed, reaching its maximum at the level of L7–S1 in all cats. Overall, the resulting trend line shows a bimodal pattern, characterized by an initial ascent, followed by a descent and subsequently, another ascent (Figure 5B).

**Figure 6:**
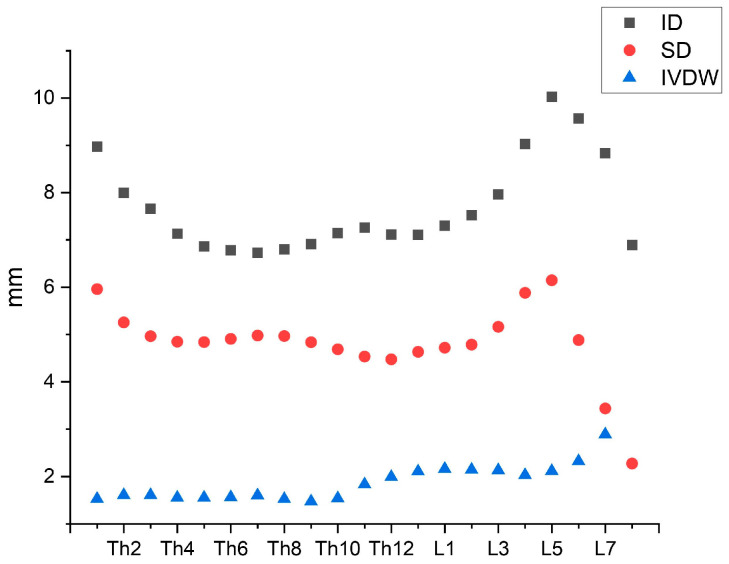
Combining illustration of interpedicular (ID) and midsagittal diameter (SD) as well as intervertebral disc width (IVDW) from Th1 to S1. The parameters are shown as mean values.

Figure 6 illustrates that ID and SD show a declining trend from Th1 to Th4. Moreover, a distinct decrease in both parameters starting from L5 is visible, whereas IVDW ascends continuously and peaks just before the vertebral canal diameters reach their minimum at the height of S1.

### 3.3. Statistical Analysis

In total, ID and SD values of each vertebral body from Th1 up to S1 in 50 cats were measured. Additionally, in 28 of the cats, IVDW was determined in the stated area. Mean values ± sd are illustrated in Table 1A, respectively Table 2A.

Table 1B and Table 2B show median values and ranges. The Shapiro–Wilk test was used to check each vertebra or intervertebral disc for normal distribution. This showed that with regard to ID, the values from vertebral bodies Th1 to L5 are not normally distributed, whereas, in terms of SD, this applies to vertebrae Th3, Th4, L1, L3 and L5. Concerning the intervertebral discs, the data from discs Th6–Th7, Th9–Th10 and Th11–Th12 exhibit signs of a non-normal distribution. The vertebrae, respectively, intervertebral discs not mentioned follow a normal distribution. Due to the inconsistency concerning the normal distribution of the values, both median and range, as well as mean ± sd, are presented.

Consequently, a Spearman rank test was conducted to establish correlations between the three parameters using mean values. For ID and SD, the respective vertebral body was set in relation to the adjacent intervertebral disc, for example, Th1 to disc Th1-Th2, and so on. A moderate, positive correlation was identified between both vertebral canal diameters ID and SD (r_s_ = 0.35; *p* = 0.12). Concerning ID and IVDW, a strong correlation was evident with r_s_ = 0.55 (*p* = 0.01). A low, negative correlation was noted between SD and IVDW (r_s_ = −0.21; *p* = 0.37).

Since the vertebral canal was not accessible for the measurements in all cases, there was a variable number of fully measured and thus analyzable animals. With regard to ID, SD and IVDW, the range from Th1 up to S1 was considered in its entirety as well as separately for the thoracic and lumbar spine. The smallest as well as the three largest values were evaluated for each parameter.

#### 3.3.1. ID

Concerning ID, complete data from Th1 to S1 were available and analyzed for 33 cats. Furthermore, thoracic spine data was analyzed for 43 and lumbar spine data for 35 cats. When considering the entire range from Th1 up to S1, in 33.33% of cats (*n* = 11) the narrowest point was detected at the level of S1. In 12.12% of cats (*n* = 4), the smallest value could be found at the vertebrae Th6, Th7, Th8, or Th9. The latter also becomes apparent when considering the thoracic spine separately. In this case, the narrowest point was determined at the level of Th7 in 27.91% of cats (*n* = 12), followed by Th6 with 20.93% (*n* = 9) and Th8 and Th9, each with 13.95% (*n* = 6). The narrowest part in the lumbar spine was detected at S1 in the majority of cats (54.29%; *n* = 19), followed by L1 with 34.29% (*n* = 12). The three widest points were found at the height of Th1, Th2, L5 and L6 in 36.36% (*n* = 12) and from L5 to L7 in 30.30% of cats (*n* = 10) when considering the range from Th1 to S1. With regard to the thoracic spine, the widest part was determined from Th1 to Th3 in 39.53% (*n* = 17) and at the level of Th1, Th2 and Th13 in 16.28% of cats (*n* = 7). In the lumbar spine, 45.71% of cats (*n* = 16) showed their three maximum values between L5 and L7 and between L4 and L6.

#### 3.3.2. SD

Regarding SD, the complete range from Th1 to S1 was available for analysis in 30 cats, whereas a total of 40 cats were included for thoracic spine analysis and 35 cats for the lumbar spine. Both when considering the entire section and the lumbar spine separately, the narrowest point was identified at S1 in all cats. Relating to the thoracic spine, the minimum could be found at the level of Th11 in 22.50% (*n* = 9) and at Th12 in 20.00% of cats (*n* = 8). Concerning the range from Th1 to S1, the three widest points were detected at Th1, L4 and L5 in most of the cats (63.33%; *n* = 19). The section between Th1 and Th3 represented the widest part with regard to the thoracic spine in 25.00% of cats (*n* = 10), followed by Th1, Th6, Th7 and Th8 with 15.00% (*n* = 6). In the lumbar spine, the three maximum values were determined between L3 and L5 in 57.14% (*n* = 20) and between L4 and L6 in 12/35 cats (34.29%).

#### 3.3.3. IVDW

When looking at IVDW, both regarding the entire range from Th1 to S1 and the thoracic spine in isolation, the narrowest intervertebral disc space was found at the level of disc Th8–Th9 and Th9–Th10, each with 30.43% (*n* = 7). In the lumbar spine, the intervertebral disc between L4 and L5 was identified as the narrowest in 10/23 cats (43.48%), followed by L5–L6 with 26.09% (*n* = 6). The widest disc was detected at the level of L7–S1 in 22/23 cats (95.65%), when considering the section from Th1 to S1. This is also reflected in the lumbar spine, where the maximum value was found at the mentioned disc in all cats. With regard to the thoracic spine, the widest disc was identified at the height of Th13–L1 in the majority of cats (69.57%; *n* = 16), followed by the disc between Th12 and Th13 with 30.43% (*n* = 7).

## 4. Discussion

Hypertrophy of the articular processes has been mentioned as the primary cause of stenosis of the feline vertebral canal in the thoracic region, which is only marginally described in the literature [18]. In the course of this work, it was found that additional reductions in the vertebral canal diameters from Th1 to Th4 (both ID and SD), from Th6 to Th7 and Th11 to Th13 (ID) as well as in the range from Th9 to Th12 (SD) can be noted. These may be further predisposing factors for the development of myelopathy. Compared to the already extensively described lumbosacral junction in dogs, it is clear that a significant lumen reduction in the area L6–S1 is also evident in cats. Therefore, in addition to myelopathies caused by trauma or neoplasia [21], the anatomical conditions in this region must be taken into account. While LSS is associated with degenerative alterations in dogs [14], similar changes could not be detected in the examined cats. The areas showing an increase in both diameters were analogically documented in dogs and corroborate the cervical and lumbar enlargements described in the literature. The cervical enlargement extends from spinal cord segment C6 to Th2, which approximately corresponds to vertebra C6 to Th2, since in cats the cranial spinal cord segments correlate strongly with the according vertebral bodies [3]. Although no measurements were taken in the cervical region in this study, the initial segments of the thoracic vertebral canal correlate with the cervical enlargement. The lumbosacral enlargement reaches from spinal segments L4 to S3 and from vertebra L4 to L6, which coincides with the findings of this study [3,22]. In the literature, a further thoracal enlargement has been mentioned in cats which is located at the level of the 12th thoracic vertebra [5]. However, this thoracal enlargement is not seen in all cats [23]. Within the scope of this study, no evidence of this enlargement could be found.

Regarding the statistical analysis, the inconsistency regarding the normal distribution of the values for the respective vertebrae or intervertebral discs could be related to differences in age, size, or sex of the animals. Considering that the cats were donated anonymously from various veterinary practices and had been previously used for educational purposes, the sex of the animals could no longer be identified during the examinations. Moreover, the present study serves as fundamental research and a significant difference between male and female animals was not anticipated. Further investigations are planned to evaluate the impact of sex. When looking at the correlative analysis and using a *p*-value < 0.05 as statistically significant, it can be deduced that there is no significant correlation between the variables ID and SD or SD and IVDW. However, the correlation between ID and IVDW is statistically significant. The latter suggests that with increasing ID, IVDW also increases. Interestingly, this condition does not apply to SD and IVDW, where a negative correlation was found. This implies that as the value of one variable increases, the value of the other variable tends to decrease. The *p*-value of 0.37 indicates that this correlation is not statistically significant. While the correlation coefficient of 0.35 suggests a moderate strength of association between ID and SD, the lack of statistical significance means that we cannot confidently conclude that this relationship exists within the examined population. Further investigation with a larger sample size may be needed to confirm or refute the observed correlations.

It can be summarized that the vertebral canals of cats show similarities to those of dogs. In both species, lumbosacral stenosis is seen, but in the cat, compared to the dog, it rarely results in clinically relevant alterations. A possible cause could be differences in the anatomical position of the spinal nerve roots as a result of the stronger relationship between spinal cord segments and corresponding vertebral bodies in caudal orientation in the cat compared to the dog. Consequently, they are less prone to compression or irritation, even in the presence of lumbosacral stenosis in cats. Likewise, a less distinctive correlation between spinal cord and vertebral canal diameter and thus, a lower tendency to spinal cord compression in the cat can be considered. Furthermore, it is essential to differentiate between absolute and relative vertebral canal stenosis. While absolute vertebral canal stenosis directly leads to compression of spinal cord structures, relative stenosis initially does not affect neural elements [11]. However, the latter is associated with a decreased space capacity of the vertebral canal, so even small vertebral deformities and age-related formations can cause spinal cord compression with consecutive clinical signs [24]. In cats, degenerative vertebral alterations analogous to those in dogs have been described [25]. One case shows vertebral canal stenosis in a cat with clinical signs of spinal cord compression due to dural ossification [26]. In addition, neurological deficits due to idiopathic skeletal hyperostosis and lumbosacral osteochondrosis have been described in cats with the latter causing symptoms of CES [27,28]. In another study, abnormalities of the lumbosacral vertebrae were stated as the cause of gastrointestinal disorders such as megacolon and constipation [29], which emphasizes not only the neurological but also the internistic relevance of vertebral canal stenoses in cats.

The cases mentioned above show once again that vertebral canal stenosis with subsequent spinal cord compression, such as CES, is also clinically relevant in cats. However, what is interesting is that cats present much less frequently with neurological signs compared to dogs [17]. Regarding the aspect of relative vertebral canal stenosis, it can be suggested that the cat’s vertebral canal has fewer or rather less pronounced relative stenoses than those of the dog. Consequently, the spinal cord would have more space capacity in the vertebral canal of cats compared to dogs, which could explain the differences in the occurrence of clinical symptoms. Prospective studies are necessary to investigate this hypothesis.

The new information regarding the IVDW presented in this study shows analogies to the data obtained in the dog. One study found that in dogs, the intervertebral discs in the thoracic spine are rather narrow and almost equally wide [9]. The narrowest point represents the intervertebral disc at the level of Th10–Th11. Subsequently, the width increases continuously until it reaches its maximum in the area of the lumbosacral junction [9]. Similar findings could also be made in the cat in this study. However, instead of a continuous increase, the cat’s IVDW shows a bimodal course starting from Th10–Th11 (Figure 5B). In this regard, it should be considered that IVDW may also depend on the animal’s age. For the dog, it has been established that the width grows with increasing age [30]. A similar relation cannot be ruled out for the cat, as age was not taken into account in this work. Moreover, since the measurements were performed with an analog caliper and the widths may vary due to the freezing and defrosting process, the values are subject to a certain degree of inaccuracy. Nevertheless, when evaluating the photographic documentation of the cats in terms of IVDW, it is conspicuous that the subjective findings correlate with the absolute values obtained. By comparative consideration of adjacent intervertebral discs, it can be assumed that the width remains relatively invariable up to the level of the thoracolumbar junction. A subsequent increase is evident in all photos, resulting in a peak at the lumbosacral junction. Further measurements of the intervertebral disc widths are indicated to confirm respectively specify the findings of this study.

Disc herniations occur rather rarely in cats, whereas they represent the majority of spinal diseases in dogs [31,32,33,34]. The reason for this could be the absence of chondrodystrophic breeds in the cat [35]. The predisposing factors are often multifarious and include age, sex and breed of the animals, among others [34]. While disc herniations do occur in cats, they rarely become clinically visible. Nevertheless, they should be considered as a differential diagnosis in paraparetic or paraplegic cats and patients with spinal dolence [16,36,37,38]. In cats, the intervertebral discs in the cervical region seem to be involved most frequently [39]. Nonetheless, since cervical disc herniations are usually asymptomatic, their occurrence is often underestimated and they are mostly detected in post-mortem studies only [40,41]. Most affected are late middle-aged and older cats, primarily with Hansen type II protrusions [39]. However, since IVDW was not measured in the cervical region in this study, no correlations can be inferred regarding this matter. When focusing on clinically relevant disc herniations, a common localization described in the literature is between L7 and S1 [34] and the intervertebral disc between L6 and L7 [41]. One study mentions the thoracolumbar junction as the most common region for clinically significant disc herniations, with middle-aged cats, in particular, being affected by Hansen type I extrusions [39]. A case report also describes a lumbar foraminal intervertebral disc extrusion in a cat with acute onset of pelvic limb lameness [42].

With a view to possible predilection sites for disc herniations, it has already been shown in studies involving Doberman Pinscher dogs that an enlarged disc width could be a risk factor for clinically relevant disc herniation [43]. According to this, the increased volume of disc material could consequently lead to a more significant spinal cord compression. In particular, a relatively narrow vertebral canal combined with a large disc width seem to be strong predisposing factors. In cats, such a combination can be found in the area of the lumbosacral junction according to the findings of this study. This can be further underpinned by the identified negative correlation between SD and IVDW, although the association was considered not statistically significant. Moreover, this condition is also evident when considering Figure 6. While ID and SD exhibit slight variances in the thoracic spine, they show a similar trend in the lumbar spine with a distinct decline in both parameters starting from L5. However, IVDW represents an opposite trend in the area of the lumbosacral junction by increasing and finally reaching a maximum at disc L7–S1 (Figure 6). These results, in association with the described trends in the cat’s disc width, could support the statements of the mentioned study [43]. Accordingly, regions with increased disc width, especially the caudal lumbar spine, seem to have an elevated risk of clinically relevant disc herniations.

Furthermore, it is conspicuous that cats of the British Shorthair and Persian breeds appear to be over-represented regarding thoracolumbar disc herniations [44]. Since British Shorthair cats also seem to be prone to thoracic vertebral canal stenosis [11], a correlation between these two conditions can be assumed. Moreover, male neutered and purebred cats seem to be predisposed to feline intervertebral disc disease [35]. Consequently, it can be speculated that purebred cats, especially British Shorthairs and Persians, are more frequently affected by pre-existing relative vertebral canal stenosis, similar to dogs. Whether this condition possibly does not apply to European Shorthair cats, and they are therefore less frequently affected by clinically relevant compressive myelopathies, needs to be clarified in further studies.

From a clinical–practical perspective, it can be assumed that spinal diseases, such as CES or disc herniations, are often underestimated in cats. These conditions may appear as incidental findings in imaging diagnostics, such as MRI or CT, without obvious clinical signs in the affected cat. Therefore, awareness of such pathologies in cats should be increased among veterinarians to expedite diagnostics and treatment in cases such as reluctance to move or lumbosacral pain and to include these conditions in the list of differential diagnoses.

## 5. Conclusions

This study presents initial results on the dimensions of the feline vertebral canal and intervertebral discs. Additionally, it provides insights into potential predilection sites for compressive myelopathies in cats. To conclude, it can be suggested that a relatively stenotic vertebral canal is a risk factor for developing clinically relevant CES and disc herniations, especially if these are accompanied by degenerative vertebral alterations, such as spondylosis or osteophytes, as well or rather in combination with areas of the vertebral canal that show an increased IVDW. In both cats and dogs, such a configuration is evident at the lumbosacral junction. Further studies on cats should be conducted to investigate the relationship between vertebral canal diameter and IVDW with consideration of age, sex and breed as well as to evaluate the hypothesis put forward.

To the authors’ best knowledge, this study is the first description of the normal feline vertebral canal dimensions and IVDW from Th1 to S1. The findings presented serve as a basis for further investigations and allow conclusions regarding possible etiologies of feline myelopathies.

## Figures and Tables

**Table 1:** Interpedicular (ID) and midsagittal diameters (SD) in 50 cats for the vertebrae Th1 up to S1. (**A**) Data are presented as mean ± sd. (**B**) Data are presented as median (md) and range (rg).

(A)			(B)				
Vertebral Body	ID (mm)	SD (mm)	Vertebral Body	ID (mm)		SD (mm)	
Th1	8.97 ± 0.97	5.96 ± 0.53	Th1	md	rg	md	rg
				9.10	3.95	6.00	2.45
Th2	7.99 ± 0.81	5.25 ± 0.52	Th2	7.90	4.10	5.18	2.38
Th3	7.66 ± 0.86	4.96 ± 0.56	Th3	7.53	4.90	5.03	2.45
Th4	7.13 ± 0.89	4.85 ± 0.61	Th4	7.05	4.78	4.90	2.60
Th5	6.86 ± 1.02	4.84 ± 0.58	Th5	6.63	7.15	5.00	3.03
Th6	6.78 ± 1.00	4.91 ± 0.56	Th6	6.65	7.20	4.94	2.95
Th7	6.73 ± 1.15	4.98 ± 0.50	Th7	6.63	8.45	5.00	2.73
Th8	6.80 ± 0.92	4.96 ± 0.58	Th8	6.70	6.55	5.00	3.13
Th9	6.91 ± 0.90	4.83 ± 0.51	Th9	6.70	5.50	4.91	2.98
Th10	7.14 ± 0.87	4.69 ± 0.49	Th10	7.05	5.00	4.70	1.83
Th11	7.26 ± 0.93	4.53 ± 0.46	Th11	7.13	6.00	4.50	2.18
Th12	7.11 ± 0.92	4.47 ± 0.55	Th12	7.13	6.10	4.51	2.53
Th13	7.10 ± 1.07	4.63 ± 0.61	Th13	6.85	7.20	4.75	2.53
L1	7.30 ± 0.95	4.72 ± 0.61	L1	7.25	5.60	4.85	2.43
L2	7.52 ± 0.91	4.78 ± 0.62	L2	7.50	5.80	4.83	2.38
L3	7.96 ± 0.98	5.15 ± 0.57	L3	7.93	5.85	5.28	2.93
L4	9.03 ± 1.13	5.88 ± 0.59	L4	8.80	7.25	5.80	2.88
L5	10.03 ± 0.90	6.15 ± 0.68	L5	9.90	4.75	6.33	2.70
L6	9.57 ± 0.85	4.88 ± 0.63	L6	9.65	3.40	4.83	2.73
L7	8.83 ± 1.34	3.43 ± 0.59	L7	8.78	5.15	3.41	2.50
S1	6.89 ± 1.17	2.27 ± 0.33	S1	7.05	4.95	2.24	1.33

**Table 2:** Intervertebral disc widths (IVDW) for discs Th1–Th2 up to discs L7–S1 in 28 cats. (**A**) Data are presented as mean ± sd. (**B**) Data are presented as median (md) and range (rg).

(A)		(B)		
Intervertebral Disc	IVDW (mm)	Intervertebral Disc	IVDW (mm)	
Th1–Th2	1.53 ± 0.32	Th1–Th2	md	rg
			1.55	1.15
Th2–Th3	1.60 ± 0.32	Th2–Th3	1.55	1.40
Th3–Th4	1.61 ± 0.41	Th3–Th4	1.50	1.50
Th4–Th5	1.55 ± 0.33	Th4–Th5	1.55	1.15
Th5–Th6	1.55 ± 0.28	Th5–Th6	1.50	1.10
Th6–Th7	1.56 ± 0.28	Th6–Th7	1.50	1.15
Th7–Th8	1.60 ± 0.27	Th7–Th8	1.55	1.10
Th8–Th9	1.52 ± 0.31	Th8–Th9	1.50	1.35
Th9–Th10	1.47 ± 0.29	Th9–Th10	1.40	1.40
Th10–Th11	1.54 ± 0.29	Th10–Th11	1.50	1.00
Th11–Th12	1.83 ± 0.35	Th11–Th12	1.80	1.05
Th12–Th13	1.99 ± 0.38	Th12–Th13	1.95	1.30
Th13–L1	2.10 ± 0.40	Th13–L1	2.00	1.40
L1–L2	2.16 ± 0.38	L1–L2	2.10	1.20
L2–L3	2.14 ± 0.38	L2–L3	2.15	1.40
L3–L4	2.13 ± 0.42	L3–L4	2.10	1.90
L4–L5	2.03 ± 0.44	L4–L5	2.00	1.60
L5–L6	2.11 ± 0.36	L5–L6	2.20	1.20
L6–L7	2.32 ± 0.49	L6–L7	2.40	1.75
L7–S1	2.88 ± 0.52	L7–S1	3.00	1.60

## Data Availability

Data can be made available upon request to the corresponding author.
